# Carbon nanotubes for flexible batteries: recent progress and future perspective

**DOI:** 10.1093/nsr/nwaa261

**Published:** 2020-10-20

**Authors:** Sheng Zhu, Jian Sheng, Yuan Chen, Jiangfeng Ni, Yan Li

**Affiliations:** Beijing National Laboratory for Molecular Sciences, Key Laboratory for the Physics and Chemistry of Nanodevices, State Key Laboratory of Rare Earth Materials Chemistry and Applications, College of Chemistry and Molecular Engineering, Peking University, Beijing 100871, China; Beijing National Laboratory for Molecular Sciences, Key Laboratory for the Physics and Chemistry of Nanodevices, State Key Laboratory of Rare Earth Materials Chemistry and Applications, College of Chemistry and Molecular Engineering, Peking University, Beijing 100871, China; School of Chemical and Biomolecular Engineering, The University of Sydney, Sydney 2006, Australia; School of Physical Science and Technology, Center for Energy Conversion Materials & Physics (CECMP), Jiangsu Key Laboratory of Thin Films, Soochow University, Suzhou 215006, China; Light Industry Institute of Electrochemical Power Sources, Suzhou 215699, China; Beijing National Laboratory for Molecular Sciences, Key Laboratory for the Physics and Chemistry of Nanodevices, State Key Laboratory of Rare Earth Materials Chemistry and Applications, College of Chemistry and Molecular Engineering, Peking University, Beijing 100871, China

**Keywords:** carbon nanotube, flexible battery, electrochemical energy storage, wearable electronics

## Abstract

Flexible batteries, which maintain their functions potently under various mechanical deformations, attract increasing interest due to potential applications in emerging portable and wearable electronics. Significant efforts have been devoted to material synthesis and structural designs to realize the mechanical flexibility of various batteries. Carbon nanotubes (CNTs) have a unique one-dimensional (1D) nanostructure and are convenient to further assemble into diverse macroscopic structures, such as 1D fibers, 2D films and 3D sponges/aerogels. Due to their outstanding mechanical and electrical properties, CNTs and CNT-based hybrid materials are superior building blocks for different components in flexible batteries. This review summarizes recent progress on the application of CNTs in developing flexible batteries, from closed-system to open-system batteries, with a focus on different structural designs of CNT-based material systems and their roles in various batteries. We also provide perspectives on the challenges and future research directions for realizing practical applications of CNT-based flexible batteries.

## Teaser

Carbon nanotubes with diverse macroscopic structures and distinctive physical, chemical, and mechanical properties are promising candidate materials for flexible batteries.

## INTRODUCTION

**Figure 1.**
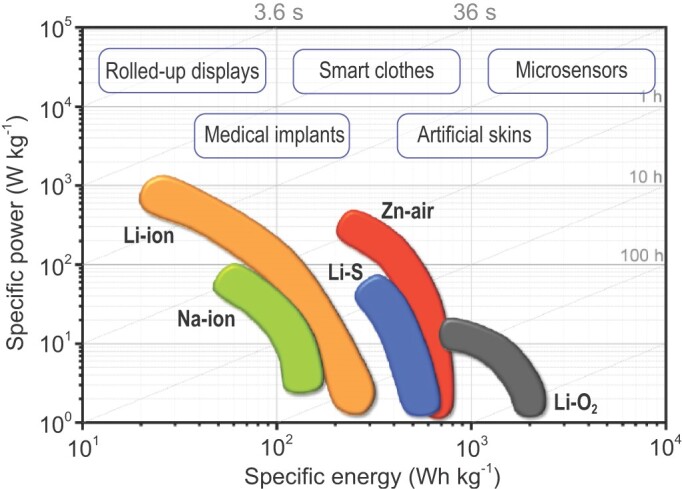
Ragone plots of diverse batteries and the commonly used electronics powered by flexible batteries.

Rechargeable batteries, which store electrical energy by reversible Faradaic reactions, are dominant energy storage devices on the current market [1–3]. They can be roughly classified into two categories [4]: open-system batteries and closed-system batteries. Open-system batteries can be defined as battery systems that can freely exchange materials and energy with their surrounding environment. Metal-air batteries, such as zinc-air batteries (ZABs) and lithium-air batteries (LABs) are representative examples. Their working mechanisms involve metal dissolution/deposition on anodes inside batteries and oxygen reduction reaction (ORR) and oxygen evolution reaction (OER) on cathodes with mass exchanges of oxygen or air with their external environment [5,6]. Owing to the minimized mass and volume of the air electrode, metal-air batteries offer higher energy density. In contrast, closed-system batteries are battery systems where only energy can be transferred without mass exchange. Representative examples include lithium-ion batteries (LIBs), sodium-ion batteries (SIBs) and lithium-sulfur (Li-S) batteries. Closed-system batteries, especially LIBs, are more widely used in practical applications due to their long cycle life, high energy efficiency and matured manufacturing technologies. Recently, the emerging portable and wearable electronics, such as rolled-up displays, smart clothes, microsensors, medical implants and artificial skins (Fig. 1), demand flexible energy storage devices [7–9]. The market of flexible batteries is forecasted to grow rapidly from $69.6 million in 2015 to $958.4 million by 2022 [10]. However, the manufacturing of commercial batteries generally depends on casting slurries of electrode materials on metal current collectors. The resulting batteries are heavy, bulky, and rigid, which cannot satisfy the surging demands from portable and wearable electronics. To this end, it is urgent to develop flexible batteries with both superior mechanical properties and electrochemical energy storage properties under various deformative conditions.

Flexible batteries need to undergo frequent mechanical deformations, such as bending, folding, twisting and stretching [11,12]. They may face two types of mechanical deformations: elastic or plastic deformations [13]. Elastic deformation is defined as a change in material shape that is recoverable after the removal of external stress, while plastic deformation refers to an irreversible change. Elastic deformation is desired for flexible batteries, and thus, they can work appropriately during and after reversible deformations without electrochemical performance degradation.

**Figure 2.**
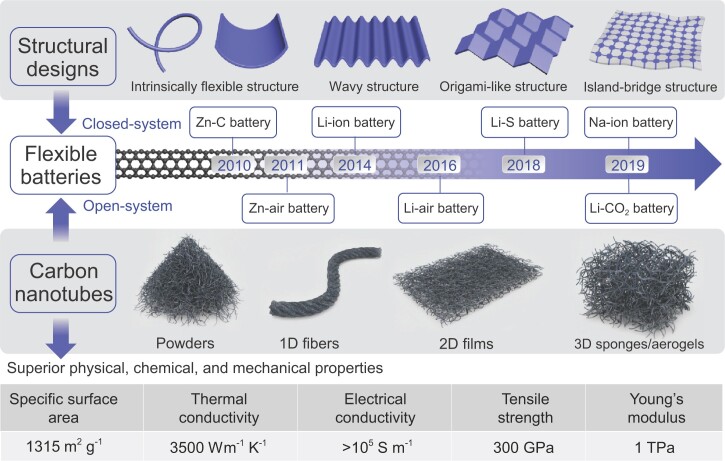
A brief chronology of the development of flexible full batteries containing carbon nanotubes.

The flexibility of batteries may be achieved by either using intrinsically deformable materials or specially-designed structures (Fig. 2). Various intrinsically deformable materials have been used to realize flexible batteries at the individual component level [13,14]. Brittle and stiff materials used in conventional batteries can also be used in flexible batteries via proper architectural designs. Four types of structures have been explored for this purpose, including (i) intrinsically flexible structures of low-dimensions, such as thin one-dimensional (1D) fibers/cables and 2D thin films; (ii) wavy/wrinkled structures by depositing stiff materials on pre-strained elastomeric substrates (e.g. polydimethylsiloxane (PDMS), Ecoflex and cotton textiles); (iii) origami-like structures by predefined crease patterns; and (iv) serpentine island-bridge structures, in which rigid materials (islands) are interconnected by bridges with high conductivity and flexibility [15,16]. It should be noted that in addition to mechanical deformability, high electrical conductivity or suitable chemical/catalytic activities are essentially required for various components in flexible batteries. Besides, the choice of electrolyte is also important for flexible batteries. Liquid electrolytes have been widely used in conventional batteries. However, they are not suitable for flexible batteries, because leakage may happen during repeated deformations, leading to performance degradation [17]. Hence, solid-state electrolytes are favorable for flexible batteries, which not only prevent the internal short-circuiting but also improve the mechanical property of the flexible batteries.

**Figure 3.**
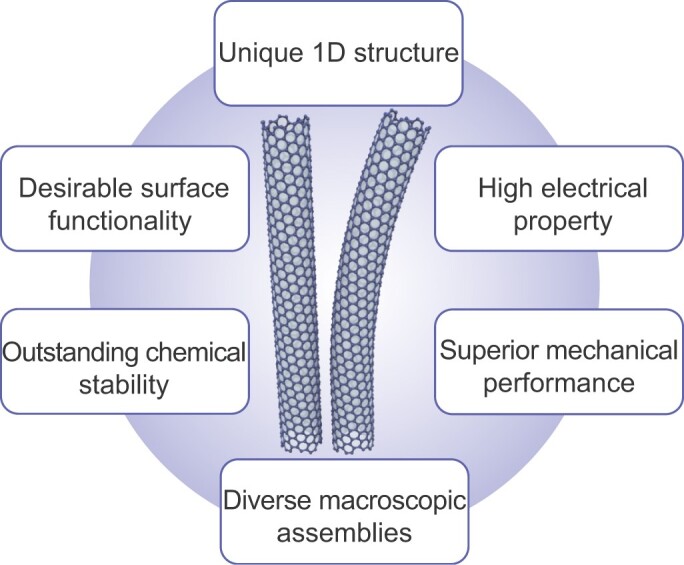
Key advantages of CNTs for flexible batteries.

Since Iijima's pioneering study in 1991 [18], carbon nanotubes (CNTs) have attracted significant interest due to their distinctive structures and properties. CNTs have been a key pioneer and constant driving force for the fast progress in nanomaterials and nanotechnology over the last three decades [19,20]. An individual CNT can be considered as a seamless cylinder formed by rolling up graphene sheets. Depending on the number of graphene sheets in their tube walls, CNTs can be categorized as single-walled CNTs (SWCNTs) or multi-walled CNTs (MWCNTs) with an interlayer distance of ∼0.34 nm between two adjacent graphene layers. CNTs are constructed with sp^2^ bonded carbon atoms, endowing individual CNTs with unique mechanical, physical and chemical properties [21]. For example, CNTs have an electrical conductivity of 10^6^ S m^−1^ for SWCNTs or over 10^5^ S m^−1^ for MWCNTs at 300 K, thermal conductivity of 3500 Wm^−1^ K^−1^, Young's modulus of 1 TPa, tensile strength up to 300 GPa, a large specific surface area up to 1315 m^2^ g^−1^, and low mass density [22–24]. The outstanding mechanical and physical property and stability of CNTs make them excellent candidate materials for flexible batteries. After tremendous effort, mass production of CNTs (in powders or liquid dispersions) has now been achieved by chemical vapor deposition (CVD). CNTs have been studied for many applications, such as energy storage, catalysis, electronics and functional composites [25–28]. They have been considered as an essential building block to create various flexible composite structures. A variety of commercial products containing CNTs are already on the market. For example, they have served as conductive additives in many batteries, which replace conventional conductive additives based on graphite or carbon black particles. More intensive application of CNTs in flexible batteries is expected in the near future.

In this review, we first discuss the unique merits that CNTs can offer for flexible batteries. Next, we highlight the recent progress of applying CNTs in various types of flexible batteries, including both closed-system and open-system batteries. Our discussion focuses on the material synthesis, fabrication of various battery components and structure design of batteries using either individual CNTs or their macroscopic assembles or composites. Last, we provide our perspectives on challenges and future research directions on using CNTs to realize flexible batteries in practical applications.

## ADVANTAGES OF USING CNTs IN FLEXIBLE BATTERIES

Laser ablation, arc discharge and CVD methods have been extensively developed for the controllable preparation of CNTs. Because of the high energy input by a laser beam or an arc discharge, the prepared CNTs demonstrate a high degree of graphitization. Nevertheless, harsh vacuum conditions and continuous graphite target replacement are required [29]. In the CVD process, the carbon sources are catalytically converted into tubular CNTs at the catalytic sites. The CVD route has the main merits of synthetic conditions (such as moderate temperature and atmospheric pressure), high yield and low cost. Moreover, the CNT structure (e.g. wall number, length, diameter and alignment) can be well controlled [20].

Due to their distinctive physical, chemical and mechanical properties (Fig. 3), CNTs have several potential advantages, which may play critical roles in realizing high-performance flexible batteries.

- CNTs have high electrical conductivity with a large aspect ratio (the length-to-diameter ratio is >10 000), which enables more efficient electron transfer compared to conventional conductive additives. Thus, they can serve as suitable conductive ingredients to form composite electrodes with less conductive electrode materials in flexible batteries.
- They have a unique 1D nanoscale structure, which can be used to create diverse nanoscale architectures to be employed in different components of flexible batteries. For example, they can work as linkers to integrate various 1D nanowires or 0D nanoparticles into nanohybrid materials. Alternatively, CNTs may serve as nanoscale spacers to prevent the stacking of 2D nanosheets, resulting in nanocomposites with high electrolyte accessible surface area.
- Due to their unique 1D structures, high aspect ratio and suitable surface chemical properties, CNTs can be assembled into different macroscopic structures, such as 1D fibers, 2D films, 3D sponges, aerogels and foams, by a variety of methods, including wet/dry spinning, vacuum filtration, self-assembly and direct CVD. Some of these macroscopic structures are highly flexible with superior mechanical and electrical properties. For example, 1D CNT fibers spun from CNT forests show an electrical conductivity of 300 S cm^−1^ with a tensile strength of 460 MPa [30]. 2D CNT films have an electrical conductivity of 2000 S cm^−1^ with a tensile strength over 360 MPa [31]. 3D CNT sponges with high porosity and controlled orientation can endure compressive stress of 0.032 MPa at the maximum strain (ϵ = 50%) [32]. These CNT macroscopic structures can be used to construct various components in flexible batteries.
- Pristine CNTs have a high degree of graphitization. The lack of surface dangling bonds leads to outstanding chemical stability in various electrolytes, which can help to broaden operating windows and improve the cycling performance of flexible batteries. On the other hand, upon suitable functionalization, CNTs provide suitable surface chemical properties required in some flexible batteries. For example, some open-system batteries require active catalysts to catalyze oxygen redox reactions. CNTs doped with heteroatoms, such as nitrogen and sulfur, have shown high catalytic activities. Furthermore, other metal particles or metal composite-based catalysts can be anchored on functionalized CNTs to form hybrid catalysts for oxygen redox reactions.
- In some batteries, for example, metal-air batteries, Faradic reactions take place at a three-phase interface, i.e. at the interface of feed gas-liquid electrolyte-solid electrocatalyst. Electrode interfaces require a balance between hydrophilicity and hydrophobicity to ensure excellent battery performance. The hydrophilicity of CNTs can be tuned by carboxylation or hydroxylation functionalization or heteroatom dopings. This surface tunability enables the creation of CNT-based electrodes with suitable interfacial properties.

## FLEXIBLE CLOSED-SYSTEM BATTERIES

Closed-system batteries are typically composed of a cathode, an anode, electrolyte, current collectors and a separator, sealed inside a casing made of packaging materials. CNTs have been widely used to fabricate various flexible current collectors and electrodes, which are essential in realizing battery flexibility. In the following four subsections, we discuss representative examples of the recent progress of CNT-based current collectors, anodes, cathodes and full cells for flexible closed-system batteries, respectively.

### Flexible current collectors

The current collector is a crucial component in batteries that enables fast electron transfer and provides mechanical support for electrode materials. Conventional batteries use aluminum or copper foils as current collectors (10–20 μm in thickness). However, they are less suitable for flexible batteries because of their heavy weight (with an areal density of 8–16 mg cm^−2^) and fast degradation under mechanical deformations resulting from weak interactions with electrode materials [33,34]. CNTs have been explored as flexible current collectors [34]. Though, high contact resistance among individual CNTs hinders electron transfer across macroscopic current collectors, compromising their performance, especially under high charging/discharging current densities. Two approaches have been reported to lower the contact resistance among CNTs in CNT current collectors. One method is to deposit a thin metal layer on CNTs [35], and the other is to use ‘epitaxial welding’ to form interconnected and crystalline structures [36].

**Figure 4.**
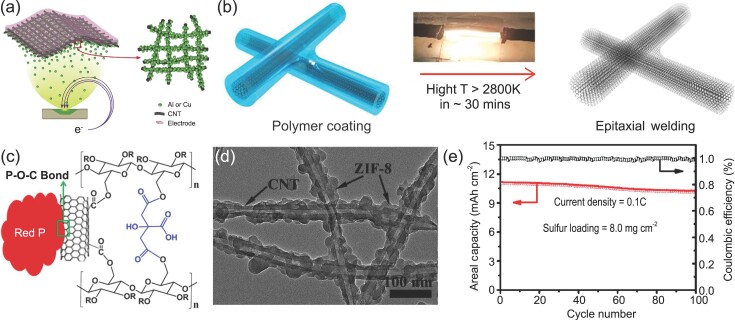
CNTs for flexible current collectors and electrodes in closed-system batteries. (a) Schematic illustration of coating super-aligned CNT current collectors with metal layers by electron-beam evaporation. Reproduced with permission from Ref. [35], copyright 2017, Elsevier B.V. (b) Schematic illustration of interconnected CNTs by ‘epitaxial welding’. Reproduced with permission from Ref. [36], copyright 2018, American Chemical Society. (c) Schematic illustration of the interactions between P, CNTs and a cross-linked polymer binder. Reproduced with permission from Ref. [44], copyright 2015, American Chemical Society. (d) Transmission electron microscopy image of the 3D CNT network grafted with MOF particles after loading sulfur. (e) Areal capacities and coulombic efficiencies over 100 charging/discharging cycles. Reproduced with permission from Ref. [53], copyright 2018, WILEY-VCH Verlag GmbH & Co. KGaA, Weinheim.

For example, thin metal films (less than 200 nm) were deposited on super-aligned CNTs by electron-beam evaporation. The resulting composite films have low sheet resistances (Al@CNT and Cu@CNT at 3.27 × 10^−1^ Ω sq^−1^ and 3.54 × 10^−1^ Ω sq^−1^, respectively). They were used as current collectors in flexible LIBs (Fig. 4a) [35]. The current collector has a thickness of <1 μm and a low areal density of 0.04 mg cm^−2^. Further, compared to other CNT current collectors fabricated using CNT liquid dispersions [37,38], the super-aligned CNTs effectively eliminate the undesired agglomeration of CNTs. The assembled flexible LIB displayed an energy density of 54.3 W h kg^−1^, much higher than the LIB assembled using Cu current collectors (18.9 W h kg^−1^). Upon folding, graphite particles adhered well with the CNT collector without performance degradation. Figure 4b shows the ‘epitaxial welding’ method [36]. The epitaxial graphitic layers effectively bridge adjacent CNTs and contribute to the high electrical conductivity of ∼1500 S/cm. It was proposed that this method can improve the electric conductivity of CNT film current collectors by one to three orders of magnitude without changing their mass and volume significantly.

However, it should be noted that many studies reported that the mass loading of active electrode materials on CNT current collectors is only ∼1 mg cm^−2^, which is 1/10 to 1/5 of the mass loading in practical batteries. The low mass loading can be attributed to the poor mechanical integrity of CNT current collectors and their weak interactions with active electrode materials. When the mass loading increases, such current collectors would break down and delaminate from active materials. Some studies have addressed this issue by using highly porous CNT films. For example, a self-supporting porous CNT macro-film (∼1 μm thick) was reported as a robust current collector for foldable LIBs [39]. Since the active materials strongly adhered to the interconnected pores, which mitigated the delamination issue, a high mass loading of 5–13 mg cm^−2^ was achieved. Impressively, the foldable LIB exhibited a high energy density of 170 Wh kg^−1^, which is approximately two times that of conventional LIBs based on metal current collectors.

### Flexible anodes

The feasibility of ion intercalation, together with excellent mechanical properties, makes CNTs attractive as anodes for flexible closed-system batteries. Early studies directly used CNT films as anodes in flexible LIBs. For instance, a free-standing CNT film was used as a flexible LIB anode without current collector and polymer [40]. However, pure CNT anodes often exhibit weak reversibility and low Coulombic efficiency. Thus, recent studies often combined CNTs with other high capacity anode materials to create flexible composite anodes [41]. However, many high capacity anode materials, such as metal alloys, inorganic compounds and polymers, are not suitable to serve as flexible anodes because of their low elasticity, poor electrical conductivity, or unstable crystalline structures. For example, Si has a thrilling theoretical capacity of (∼4200 mA h g^−1^) for LIBs, but it undergoes an excessive volumetric expansion (up to 400% during lithiation/delithiation cycling) [42]. Therefore, it is critical to design CNTs as scaffolds to mitigate these issues.

There are two different approaches to forming flexible CNT-based composite anodes: surface deposition on the CNT outer surface or incorporation into the CNT matrix. The surface deposition is beneficial for enabling easy access to electrolytes. However, due to relatively weak interactions between CNTs and anode materials, such composite anodes often show relatively low electrical conductivity, and poor mechanical and electrochemical performances, especially under high mass loadings. In comparison, incorporating high capacity anode materials into the internal space of the porous CNT matrix provides more intimate interactions between CNTs and anode materials. Further, CNTs may also efficiently restrain the extrication and suppress the volume expansion of incorporated materials. Thus, this method is more widely used in current studies.

For example, Evanoff *et al*. reported a CVD method to prepare flexible anodes by coating a uniform Si layer on CNT fabrics [43]. CNT networks can not only improve the electrical conductivity and mechanical behavior of the composite electrode but also serve as a buffer matrix to effectively accommodate the crystalline expansion and contraction of Si. Forming chemical bonds between anode materials and CNTs can further improve the structural and cyclic stability of composite anodes. For example, Son *et al*. fabricated a stable and flexible film anode for SIBs by using a chemically bonded red phosphorus-CNT hybrid, and CNTs were cross-linked with a polymer binder, as depicted in Fig. 4c [44]. Accordingly, P—O—C bonds enable high cycling stability.

### Flexible cathodes

Similar to flexible anodes, CNTs can play several beneficial roles in flexible cathodes: serving as robust substrates to provide the required mechanical flexibility, offering an electrically conductive network for less conductive cathode materials, and stabilizing some cathode materials. Many studies have focused on synthesizing robust CNT-based architectures that can host a high mass loading of active cathode materials without compromising their mechanical and electrochemical performance. For example, a flexible LiCoO_2_/CNT cathode for LIBs was prepared by a facile paper-making method with a high areal mass loading of 40 mg cm^−2^ [45]. This flexible electrode possessed a large size of 30 × 30 cm^2^ and a compressed thickness of 90 μm. LiCoO_2_ particles were uniformly embedded within the continuous 3D CNT matrix with desirable flexibility.

Among a variety of closed-system batteries, Li-S batteries are attractive because they offer high theoretical capacity (1672 mA h g^−1^) and energy density (2600 Wh kg^−1^) [46]. However, low electrical conductivity (5 × 10^−30^ S cm^−1^ at 25°C), massive volume expansion (∼79%) and dissolution of polysulfides Li_2_S_n_ (3 ≤ n ≤ 6) from the surfer cathode have limited the development of flexible Li-S batteries [19,47]. Various flexible CNT matrixes, such as CNT films [48], papers [49], sponges [50] and foams [51], have been explored to address these issues. Combining CNTs with polar metal oxides is a promising method to restrain the dissolution of polysulfide intermediates [52]. Figure 4d shows a hybrid made of CNT network grafted with small metal-organic framework (MOF) particles, serving as a flexible sulfur host [53]. Interestingly, the large MOF particles can effectively immobilize polysulfides through both physical confinement and chemical bonds, thus delivering excellent cycling stability (Fig. 4e).

### Flexible full cells

Although many studies have reported various CNT-based current collectors and electrodes for flexible batteries, fewer efforts have investigated the assembly and performance of CNT-based flexible full cells. Due to the unique properties of CNTs, they can enable various types of flexible batteries with different cell configurations. Here, we highlight three kinds of cell configurations: thin-film batteries, cable batteries and stretchable batteries.

#### Thin-film batteries

**Figure 5.**
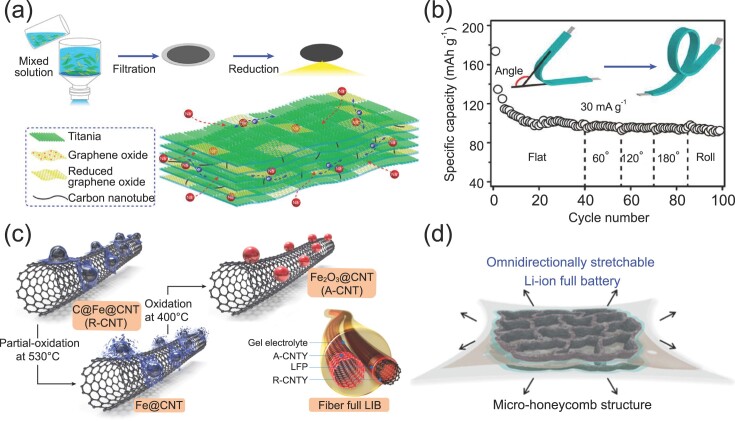
CNTs for flexible full closed-system batteries. (a) Schematic illustration of the synthesis and structure of a TiO_2_/GO/CNT hybrid electrode. (b) The specific capacity of the thin-film SIB under different bending angles. Reproduced with permission from Ref. [54], copyright 2019, American Chemical Society. (c) Schematic illustration of the synthesis of a CNT yarn anode and the assembly of cable LIB. Reproduced with permission from Ref. [58], copyright 2016, Elsevier B.V. (d) Schematic illustration of the 2D re-entrant micro-honeycomb structured electrode. Reproduced with permission from Ref. [71], copyright 2020, American Chemical Society.

As discussed above, 2D CNT thin films can enable various flexible current collectors and electrodes; thus, they have been used to assemble many bendable/foldable thin-film batteries. Usually, active electrode materials are incorporated into CNT thin films to create hybrid thin films. There are two main routes to creating hybrid CNT thin films. One is to assemble them by vacuum filtration, evaporation or self-assembly from their liquid dispersions. In some hybrid films, nanoscale CNTs can serve as useful spacers to prevent the stacking of other nanomaterials. For example, Chu *et al*. built a flexible quasi-solid-state thin-film SIB by using thin-film electrodes made of TiO_2_ nanosheets [54]. Figure 5a illustrates the synthesis of free-standing hybrid electrodes by filtration. CNTs serve as spacers to prevent the stacking of TiO_2_ and graphene oxide nanosheets. The resulting architecture can facilitate the electrolyte infiltration and ion intercalation. The assembled SIB, by coupling the TiO_2_/GO/CNT hybrid anode with a Prussian-blue-based cathode in polymer gel electrolyte, delivered cyclic stability under different deformative conditions (Fig. 5b).

Because surfactants used to disperse CNTs in liquid are often insulating materials, they may compromise the electrical conductivity of assembled CNT thin films. In addition, the aggregation of CNTs during their assembly also influences the performance of resulting CNT films. The other method to construct hybrid CNT thin films is to synthesize them by CVD directly. In some cases, the direct synthesis methods provide CNT films with appropriate orientations, high electrical conductivity and excellent mechanical strength to enable high-performance thin-film batteries. For instance, Wang *et al*. synthesized super-aligned CNT arrays by a low-pressure CVD method, which was used as a robust scaffold to embody TiO_2_ and LiMn_2_O_4_ particles [55]. A thin-film LIB was assembled using the free-standing TiO_2_/CNT anode and LiMn_2_O_4_/CNT cathode, which showed no obvious performance degradation after 500 bending cycles.

The scalable production of CNT thin films is of considerable significance for realizing practical thin-film batteries. Assembly methods via CNT liquid dispersions usually have better scalability. The complex procedures and high temperature often limit the scalability of CVD methods. Much effort has been put into addressing this issue. For example, Wu *et al*. fabricated horizontally oriented CNT films of a large size (1800 mm × 1000 mm), which showed enhanced electrical conductivity and high shape conformability by proper surface/interface engineering [56]. Highly foldable thin-film LIBs were demonstrated using such CNT films, which displayed a high capacity of 700 mAh and desirable cycling performance under folding conditions.

#### Cable batteries

1D cable structures offer high mechanical flexibility. CNTs can be assembled into continuous 1D fibers by using wet or dry spinning techniques. Thus, cable batteries based on CNT fibers with desirable electrochemical and mechanical properties have been developed. For example, Liu *et al*. fabricated a cable LIB with an ultrahigh-energy density of 215 mWh cm^−3^ [57]. CNT fibers were produced from macroscale CNT films, which served as the core of the cable in the center and were wrapped with insulating materials, anode, cathode and separator layer by layer. The CNT fiber core not only provides high electrical conductivity but also strengthens the mechanical properties of the assembled cable LIB. The cable LIB with a diameter of ∼2 mm and a length of ∼1200 mm demonstrated high flexibility. In another study, a facile oxidation step was used to convert Fe catalysts in CNT yarns to electrochemically active Fe_2_O_3_ nanoparticles [58]. As illustrated in Fig. 5c, carbon layers around Fe catalysts were eliminated by partial oxidation because of their relatively lower degree of graphitization. The resulting Fe_2_O_3_ nanoparticles were distributed homogeneously on CNT surfaces without apparent agglomeration. A cable LIB was demonstrated using the Fe_2_O_3_/CNT anode coupled with a LiFePO_4_/CNT cathode.

A unique advantage of cable batteries is that they can be further woven into flexible textiles to power lightweight and flexible wearable electronics. Peng *et al*. developed a coaxial cable LIB by sequentially scrolling two aligned CNT yarn electrodes onto a cotton fiber with gel electrolyte [59]. The aligned CNTs provide excellent mechanical and charge transport properties. The cable LIB was further woven into a flexible textile, demonstrating an areal energy density of 4.5 mWh cm^−2^. Other than cable LIBs, CNT fibers have also been used to build cable SIBs, Li-S batteries and Zn batteries [60–63].

#### Stretchable batteries

Among various mechanical deformations, such as bending, twisting and stretching, it is more challenging to achieve stretchability in flexible batteries [64]. However, it is a crucial requirement for many practical wearable devices. Two types of methods have been explored to build stretchable batteries using CNTs: coating materials on elastic substrates and creating free-standing elastic structures.

In the first method, CNTs are directly coated on elastic substrates, such as polymers or cotton textiles, to fabricate stretchable electrodes [65–67]. However, such stretchable electrodes usually have limited electron and ion transport properties, resulting in relatively poor battery performances. For example, when PDMS was used as an elastic substrate for flexible LIBs, Li ions were unable to diffuse through the PDMS scaffold. Thus, it is necessary to create Li^+^ transfer pathways in PDMS. One study reported a porosity engineering method by phase separation of polymethylmethacrylate (PMMA) in PDMS and subsequent removal of PMMA to create Li^+^ transfer pathways [68]. The resulting 3D PDMS/CNT electrodes with well-controlled pore networks show improved electrochemical and mechanical properties. The assembled flexible LIB demonstrated ∼670% higher capacity than nonporous electrodes.

The second method is to create free-standing elastic structures using CNTs as stretchable electrodes. For instance, Peng *et al*. fabricated spring-like fiber electrodes by twisting aligned CNT films, demonstrating a high elongation of up to 300% [69]. The resulting cable LIB can retain its performance upon the different bending or stretching stages. Upon an elastic stretching deformation applied to a flexible battery, the CNT electrode shows recoverable variation in spring-like structure and resistance after the release of high tensile strain [17]. However, once suffering a higher stretchability beyond 300%, the plastic stretching deformation will cause an irreversible change in structure as well as the performance decay of the flexible batteries. In another work, Peng *et al*. used helical CNT fibers to fabricate a super-stretchy LIB with a strain of 600%, which was also produced from helical CNT fibers [70]. Recently, an all-component stretchable LIB was reported based on a re-entrant honeycomb-shaped electrode, as shown in Fig. 5d [71]. The LIB displayed long-term stability over 100 cycles under the applied strain ranging from 0 to 50%. Besides, stretchable CNT electrodes with wrinkled or arched structures have also been explored in stretchable batteries [72,73].

## FLEXIBLE OPEN-SYSTEM BATTERIES

Open-system batteries are typically composed of a metal anode, electrolyte, air cathode loaded with active catalysts and other additional components, such as current collectors and gas diffusion layers. The most common open-system batteries include LABs using non-aqueous electrolytes and ZABs containing aqueous electrolytes. In this section, we discuss recent progress in using CNTs to develop flexible open-system batteries. CNTs can be used in current collectors and anodes similar to those in closed-system batteries. As for cathodes, CNTs are usually employed as conductive supports for loading catalysts to accelerate the dynamics of ORR and OER processes. Thus, we will focus on the development of CNT-based air electrodes, which are unique to open-system batteries. Based on the morphology, synthesis methods and roles played by CNTs in air electrodes, CNT-based flexible air electrodes can be categorized into four types: (i) CNT powders added as active components or conductive additives supported on non-CNT flexible substrates, (ii) CNTs grown on non-CNT flexible substrates, (iii) free-standing CNT macroscopic structures assembled using CNTs, and (iv) free-standing macroscopic CNT substrates by direct synthesis. In the following subsections, we describe these four types of CNT-based flexible air electrodes, respectively.

### CNT-containing hybrid catalysts deposited on non-CNT substrates

Many hybrid catalysts are comprised of CNTs as catalytically active components and/or conductive additives. They are synthesized by adding CNTs into metal salt precursors. The resulting hybrid catalysts in solid powders are then mixed with a binder solution to form a slurry, which is then drop-casted on non-CNT flexible substrates. The non-CNT flexible substrates provide mechanical flexibility, while the deposited catalysts deliver catalytic activities. The resulting flexible air electrodes can usually withstand simple bending and twisting mechanical deformations.

**Figure 6.**
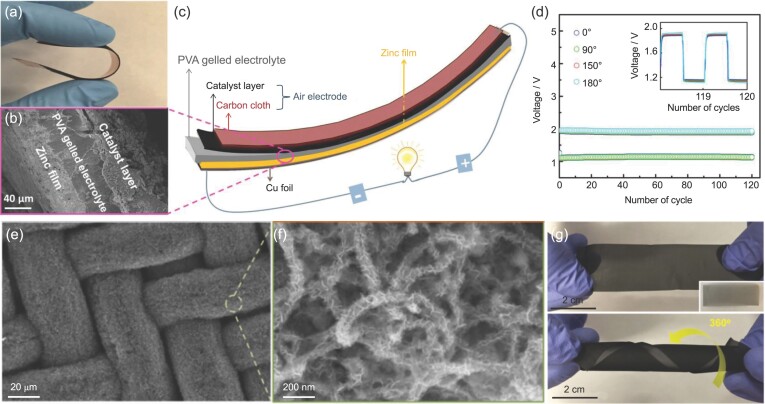
Flexible substrates supported CNTs as air electrodes for flexible open-system batteries. (a) A photo of flexible ZAB containing LaNiO_3_/NCNT as an oxygen catalyst. (b) A cross-sectional scanning electron microscopy (SEM) image of the ZAB. (c) Schematic illustration of a flexible ZAB assembled using the LaNiO_3_/NCNT cathode. (d) Galvanostatic charging/discharging pulse cycling at different bending angles. Reproduced with permission from Ref. [74], copyright 2015, WILEY-VCH Verlag GmbH & Co. KGaA, Weinheim. (e and f) SEM images of the Co_3_O_4_/NCNT catalyst supported on a stainless-steel (SS) mesh. (g) Photos of the Co_3_O_4_/NCNT/SS air electrode without and with twisting to 360°. Reproduced with permission from Ref. [79], copyright 2016, WILEY-VCH Verlag GmbH & Co. KGaA, Weinheim.

For example, Chen *et al*. synthesized the perovskite lanthanum nickel oxide (LaNiO_3_)/ nitrogen-doped CNT (LaNiO_3_/NCNT) hybrid catalyst. The catalyst was then coated on a flexible substrate made of carbon cloth as an air electrode for flexible ZAB (Fig. 6a and b) [74]. The assembled ZAB (Fig. 6c) exhibited stable discharge and charge potentials at different bending angles (Fig. 6d). NCNTs have also been used in several other studies to form hybrid catalysts with other more active catalytic components [75–77], and NCNTs not only prevent the aggregation of active catalyst particles but also create synergistic effects in hybrid catalysts. For instance, Liu *et al*. demonstrated an island-bridge design for a wearable LAB with the help of two adhesive tapes [78]. The overall electrode consists of tiny RuO_2_/CNT cathode disks and lithium anode disks interconnected by carbon ropes and copper wires, respectively. This ‘break up the whole into parts’ strategy remits the stress of electrode in the process of deformation. As a result, the discharge-charge curves kept almost unchanged even after 10 000 folding/stretching cycles.

### CNT-containing hybrid catalysts synthesized *in situ* on non-CNT substrates

Deposited hybrid catalysts may be buried inside substrates without proper access to electrolytes and air. In addition, the weak physical interactions between catalysts and substrates may also lead to catalyst detachment under mechanical deformation, resulting in poor performance flexible batteries. Alternatively, CNTs can be directly grown on non-CNT substrates, which is beneficial to facilitate charge transfer as well as mass diffusion. Besides, CNTs may form stronger covalent interactions with substrates, which reduce interface resistance and prevent catalyst detachment under mechanical deformation.

NCNTs are usually grown to provide additional catalytic activity. For example, Chen *et al*. directly grew hair-like NCNTs on a flexible SS mesh, and then coated the NCNTs with 2D Co_3_O_4_ nanopetals by electrodeposition, as shown in Fig. 6e and f [79]. Due to the excellent mechanical properties of the SS mesh, the air electrode exhibited superior flexibility, which can be twisted at a torsion angle of 360° (Fig. 6g). A flexible ZAB assembled with this air electrode delivered similar discharge and charge potentials at different bending angles. A similar strategy was also reported by Zhang *et al*. to grow NCNT on SS mesh for cable-type flexible LABs [80]. CNTs have also been grown on other carbon substrates, such as carbon cloth and electrospun carbon nanofibers (CNFs). For example, Wang *et al*. grew NCNT arrays on a carbon cloth deposited with Fe and Ni salts. The resultant composite served as air-electrodes in flexible ZABs [81]. Jiang *et al*. encapsulated Co precursors into polyacrylonitrile fibers via electrospinning and then used the fibers to catalyze the growth of CNTs (Co-N-CNT/CNF) [82]. A flexible LAB fabricated using a self-standing Co-NCNT/CNF cathode can power a red light-emitting diode display screen under different bending conditions.

### Assembled free-standing CNT electrodes

Although non-CNT flexible substrates provide necessary mechanical supports, they increase the total mass of electrodes, compromising the specific energy density of flexible batteries. Due to the strong π–π interactions among individual CNTs, they can be assembled into free-standing macroscopic structures and serve as air electrodes. Such macroscopic CNT structures provide continuous conductive paths and tunable pores for enabling electrolyte access. They can perform multiple functions in air electrodes, including current collectors, gas diffusion layers and substrates for catalysts. There are two approaches to creating such macroscopic CNT structures. One is to assemble them using individual CNTs. For example, CNT powders are dispersed in liquid solutions, and then flexible CNT films are produced by vacuum filtration [83], printing [84] and spray-coating [85]. Two different CNT films were stacked together by Srinivasan *et al*. to act as air electrodes for ZABs [86]. Notably, the hydrophobic CNT film prevented the overflow of liquid electrolyte and allowed free diffusion of oxygen, while the hydrophilic CNT film provided good electrolyte wetness.

**Figure 7.**
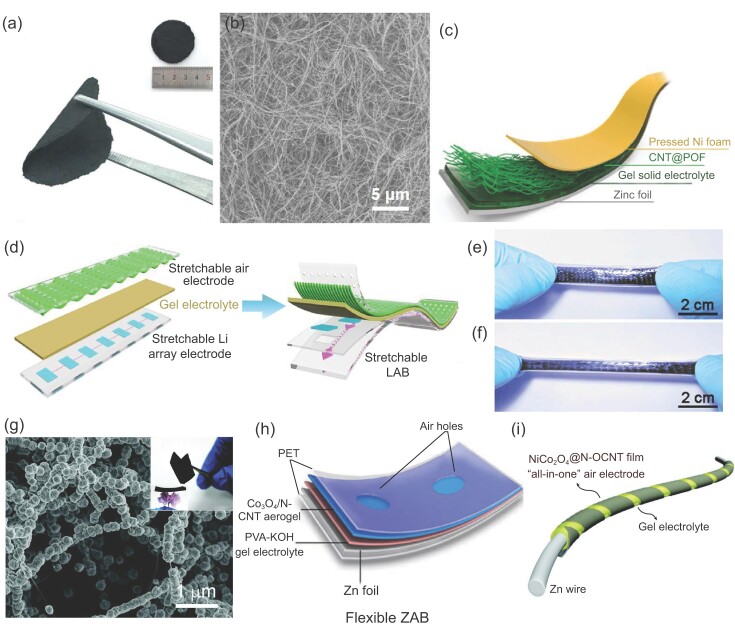
Free-standing CNT assemblies as air electrodes for flexible open-system batteries. (a) Photos of the free-standing CNT@POF film in the bent and extended (inset) conditions. (b) SEM image of CNT@POF. (c) Schematic illustration of a flexible ZAB assembled using the CNT@POF cathode. Reproduced with permission from Ref. [87], copyright 2018, The Royal Society of Chemistry. (d) Schematic illustration of the fabrication of a stretchable LAB. (e and f) Photos of the stretchable LAB before and after stretching, respectively. Reproduced with permission from Ref. [91], copyright 2016, The Royal Society of Chemistry. (g) SEM image of Co_3_O_4_/NCNT aerogel (the inset shows a photo of the flexible CNT aerogel). (h) Schematic illustration of the structure of a flexible ZAB assembled using the Co_3_O_4_/NCNT aerogel. Reproduced with permission from Ref. [94], copyright 2017, WILEY-VCH Verlag GmbH & Co. KGaA, Weinheim. (i) Schematic illustration of the structure of a flexible cable ZAB assembled using NiCo_2_O_4_@N-OCNT films. Reproduced with permission from Ref. [95], copyright 2018, WILEY-VCH Verlag GmbH & Co. KGaA, Weinheim.

Due to the relatively low catalytic activities of pristine CNTs for ORR and OER, other active catalysts are usually incorporated into CNT films. They can be added after the assembly of CNT films by dip coating, electrodeposition or sputtering. Alternatively, catalysts can be loaded on individual CNTs as hybrids first and then assembled with CNTs into CNT films. Generally, pre-assembly methods provide a more uniform distribution of catalysts on CNT films. For example, Wei *et al*. coated the porphyrin covalent organic framework on CNTs (CNT@POF) and then fabricated a free-standing film by filtration (Fig. 7a and b) [87]. The composite film was used as air electrodes for flexible ZABs (Fig. 7c), which exhibited stable discharge and charge voltages, as well as excellent cycle stability when bent to different angles.

Besides the assembly form liquid dispersions, CNT films can also be directly drawn from super-aligned CNT arrays, in which thin bundles of CNTs display unidirectional alignment [88,89]. Such CNT films are very thin, and they can be stacked layer by layer to form a conductive and porous network. Peng *et al*. fabricated a cable ZAB using such cross-stacked aligned CNT films [90]. Benefiting from the controllable cross-stacking angles of the aligned CNT films, the ZAB can be stretched to some extent without significant performance loss. Soon afterward, they also fabricated a stretchable air electrode for an LAB by stacking aligned CNT sheets on a pre-stretched polymer substrate and then releasing the substrate [91]. Figure 7d shows the air electrode with rippled aligned CNT sheets on the surface. The assembled LAB delivered a high stretchability (Fig. 7e and f), and a stable discharge voltage plateau under different tensile strains.

Assembling CNT films from liquid CNT dispersions is a low-cost and scalable method. However, the acid treatment and ultrasonication required to achieve uniform CNT dispersion affect the electrical conductivity and mechanical properties of assembled CNT films. CNT films made of cross-stacked aligned CNT sheets also show weaker mechanical strength compared to carbon cloth or metal mesh. Thus, it is desirable to further improve the mechanical properties of assembled CNT films.

### Directly synthesized free-standing CNT electrodes

The other method to create macroscopic CNT structures is to synthesize them from various liquid or gas precursors by CVD directly. The resulting structures usually demonstrate high flexibility, superior electrical conductivity, large surface area and adjustable chemical properties and more uniform morphology compared with those assembled from liquid CNT dispersions. They also have the potential to be produced in continuous large-scale manufacturing processing.

Floating catalytic CVD is the most commonly used method to grow CNTs and *in situ* assemble them into networks [32,92]. The CNT networks contain interconnected CNTs with high porosity, which provide excellent mechanical properties and facilitate the mass transport of electrolytes. For example, Zhou *et al*. used CVD-grown CNT films as binder-free air electrodes to assemble LABs [93].

CNT networks grown by CVD are usually hydrophobic. Various surface functional groups, such as hydroxyl and carboxyl, have been introduced to CNT networks to improve catalytic activities and enhance interactions with other catalysts. For instance, Li *et al*. synthesized a lightweight CNT aerogel film by a CVD method [94]. After electrochemical oxidation, Co_3_O_4_ catalysts were loaded on CNTs (Co_3_O_4_/NCNTs) (Fig. 7g). The composite film was used as air electrodes for flexible ZABs (Fig. 7h). Benefiting from the interconnected CNT networks, the assembled ZABs exhibit a small ohmic resistance of 5 Ω, and have no noticeable resistance change when folded to 90°. Using similar CNT films, they also reported a hybrid catalyst comprised of NiCo_2_O_4_ nanosheets *in situ* grown on N, O dual-doped CNTs (NiCo_2_O_4_@N-OCNT) [95]. The obtained composite films with bifunctional catalytic activities were used as air electrodes for cable ZABs (Fig. 7i).

## SUMMARY AND PERSPECTIVES

CNTs with excellent mechanical, electrical, structural and chemical properties have been shown to be superior materials for flexible batteries. CNT films and fibers can serve as lightweight flexible current collectors. Diverse CNT macroscopic assemblies, including 1D fibers, 2D films and 3D sponges and aerogels, have also found wide application as various flexible electrodes by incorporating active electrode materials or catalysts. CNT-based flexible batteries in different morphologies, including film, cable and stretchable batteries, have been demonstrated. The architecture of the batteries and the procedures of growth, assembling, functionalization and decoration of CNTs need to be optimized cooperatively to realize a satisfying performance. Although considerable advances have been achieved (Tables 1 and 2), the development of flexible batteries using CNTs is still at the early stage. The following are some of the main challenges in the development of CNT-based flexible batteries.

### High-performance CNT assemblies

Hundreds of tons of CNTs have been used in conventional battery electrodes annually as conductive additives, which has driven the rapid commercialization of CNTs at a large industrial scale [96]. The gradual maturing of CNT manufacturing technology is bound to drive down the price of CNTs further, opening the application opportunities in flexible batteries. Mechanically robust and self-supporting CNT assemblies are highly desirable for building flexible batteries. Over the last decade, a variety of CNT assemblies, ranging from 1D fibers and 2D films to 3D aerogels/foams, have been explored in flexible batteries. However, there are still many challenges in realizing their practical application.

- Enhanced mechanical and electrical properties. The mechanical strength and electrical conductivity of one single CNT are at least an order of magnitude higher than that of traditional flexible substrates, such as metal meshes and carbon cloth. However, the properties of CNT assemblies are often not satisfactory; for example, the weak interactions and large contact resistances among individual CNTs. Welding adjacent CNTs together by metal nanoparticles or adding other conductive carbon nanoparticles to form cross-linked structures may be a practical solution. However, the precise control, scalability and cost of such methods have to be carefully studied. Furthermore, some novel CNT assemblies with unique architectures, such as wavy fibers/films or helical coils, may play important roles in stretchable components in flexible batteries.
- High-density CNT assemblies. Flexible batteries need to be very thin and light, thus they ought to have a high energy density. Free-standing CNT electrodes may avoid the use of metal current collectors, which can significantly reduce the mass of batteries. However, CNT assemblies often have a large surface area, low packing density and high porosity, which results in flexible batteries with low volumetric energy density. For closed-system batteries, electrodes with a large surface area favor the electrolyte decomposition and form a highly resistive SEI-like structure, resulting in a large first-cycle irreversible capacity. Besides, increasing the density of CNTs may also allow the loading of more active materials, which is beneficial in improving the overall energy density.
- Scalable manufacturing. Despite the fact that various CNT assemblies have been demonstrated in research labs by multiple methods, the lack of scalable manufacturing methods remains a key hurdle for the practical applications in flexible batteries. For liquid-dispersion-based assembly methods, the assembly of CNTs puts forward lots of technical demands for the precise control of length to diameter ratios and surface functional groups on pristine CNTs. Dry assembly methods currently still have a very high cost. Considering further requirements on CNT functionalization to load active materials, the methods still need significant improvements.

### Controllable functionalization of CNTs

Flexible CNTs can be combined with other inflexible materials, such as metal oxide and metal alloy particles, to obtain hybrid materials with desirable mechanical and electrochemical properties. To this end, the inert surface of pristine CNTs has to be functionalized by adding heteroatoms or oxygen-containing functional groups. The functionalized surface can offer strong interactions with active materials, thus ensuring the structural stability of resulting composite materials. However, the functionalization of CNTs often induces more defects, resulting in lower electrical conductivity and mechanical properties. The chemical stability of functionalized CNTs may also degrade, especially during long-term cycling in open-system batteries. For example, OER in metal-air batteries takes place in the strong oxidative condition, which easily leads to the oxidation of unstable carbon materials. Thus, precise control in the functionalization of CNTs is a big challenge.

Besides, the functionalization method has to be able to modify CNTs with different properties at different locations precisely. For example, the uniform functionalization of CNTs is beneficial to load a large number of active electrode materials, thus increasing the overall energy density in closed-system batteries. For open-system batteries, since oxygen reactions occur at the three-phase interface, one side of the cathode needs to provide access to liquid electrolytes, and it supports catalysts, while the other side needs to be in contact with the air, and it prevents the leakage of electrolytes. Then the Janus structure, which is hydrophilic on one side and hydrophobic on the other, would be ideal. However, it is ambitious to just functionalize one side of the CNT films.

### Standardization in performance evaluation of batteries

Compared with conventional batteries, flexible batteries require performance evaluation related to both their electrochemical and mechanical properties. At present, it is still difficult to compare the results reported because diverse performance evaluation methods have been used. For example, the evaluation of electron flexibility requires a detailed description of multiple mechanical deformation conditions, such as bending, twisting and stretching. Such information is missing in many reports. For assembled flexible batteries, it is necessary to monitor their electrochemical performance under dynamic deformation conditions. After tests, electrode materials should be re-characterized to determine whether significant structural changes have taken place. Besides, when the energy density of flexible batteries is calculated, the total mass of all components should be considered rather than the mass of active electrode materials alone.

Considering that the eventual target for flexible batteries is to power wearable electronics, more attention should be paid to the integrated system. It is necessary to optimize the configuration to make different functional parts of the integrated devices function individually and match well with each other. Additionally, continuous production techniques and comprehensive performance evaluation of the integrated system also need to be developed in the future.

Although many challenges remain, recent advances have demonstrated the promising potential of CNT-based flexible batteries toward practical applications. Creating macroscopic CNT assemblies coupled with active catalysts with enhanced electrochemical and mechanical performances can push the performance limits of flexible batteries. The development of scalable production methods for CNT assemblies and flexible battery assembling techniques are vital in making flexible batteries competitive on the market. With ongoing research and development efforts, we believe that CNT-based flexible batteries will have a bright future.

**Table 1.** Performance summary of flexible closed-system batteries.

Battery device	Capacity	Energy density	Cyclability	Flexibility evaluation	Ref.
Prussian blue//TiO_2_/GO/CNT	110 mA h g^−1^, 50 mA h g_sum_^−1^ at 30 mA g^−1^	∼100 Wh kg_sum_^−1^ at 30 mA g^−1^	200 cycles at 30 mA g^−1^	Bendable	[54]
LiMn_2_O_4_/CNT array//TiO_2_/CNT array	150 mA h g^1^ at 5 C	None	100 cycles at 10 C	Bendable	[55]
LiCoO_2_/CNT film//Li_4_Ti_5_O_12_/CNT film	161 mA h g^−1^ at 0.15 A g^−1^	160 Wh Kg^−1^	100 cycles at 0.15 A g^−1^	Foldable	[56]
LiCoO_2_/CNT macrofilm//Li_4_Ti_5_O_12_/CNT macrofilm	135 mA h g^−1^ at 0.5 C	215 mWh cm_sum_^−3^	100 cycles at 0.5 C	Bendable; stretchable	[57]
Li_2_MnO_4_/CNT yarn//CNT-Si/CNT yarn	106.5 mA h g^−1^ or 0.22 mA h cm^−1^ at 148 mA g^−1^	27.7 mWh g^−1^ or 0.75 mWh cm^−1^	100 cycles at 148 mA g^−1^	Bendable; weavable	[59]
rGO/CNT/S//Li	762 mA h g^−1^ or 0.44 × 10^6^ mA h L^−1^ at 0.1 C	917 Wh L^−1^	None	Bendable	[61]
MWCNT/S//Li	1134.4 mA h g^−1^ at 0.1 C	None	100 cycles at 0.1 C	Foldable; twistable	[62]
MnO_2_/CNT fiber//Zn	290 mA h g^−1^ at 0.1 A g^−1^, 51 mA h g^−1^ at 2 A g^−1^	437 Wh Kg^−1^	100 cycles	Bendable	[63]
LiMn_2_O_4_/CNT fiber//Li_4_Ti_5_O_12_/CNT fiber	92.4 mA h g^−1^ or 2.2 mA h m^−1^ at 0.1 mA cm^−1^	None	100 cycles at 0.1 mA cm^−1^	Bendable; stretchable	[69]
LiMn_2_O_4_/CNT fiber//Li_4_Ti_5_O_12_/CNT fiber	91.3 mA h g^−1^ or 0.36 mA h m^−1^ at 0.1 mA cm^−1^	30 Wh kg_both electrodes_^−1^ or 10.6 mW h cm^−3^	100 cycles at 0.1 mA cm^−1^	Bendable; stretchable; weavable	[70]
LiFePO_4_/CNT/graphene//Li_4_Ti_5_O_12_/CNT/graphene	154 mA h g^−1^, 5.05 mA h cm^−2^, or 14.4 mA h cm^−3^ at 0.2 C	102.4 Wh kg_both electrodes_^−1^	100 cycles at 0.5 C	Stretchable	[71]
Li(Ni_1/3_Co_1/3_Mn_1/3_)O_2_/CNT//Li_4_Ti_5_O_12_/CNT	0.52 mA h cm^−2^ at 0.1 C	None	50 cycles at 0.1 C	Stretchable	[72]
LiMn_2_O_4_/CNT//Li_4_Ti_5_O_12_/CNT	0.11 mA h cm^−2^ at 175 mA g^−1^	1.6 mW h cm^−2^	100 cycles at 175 mA g^−1^	Stretchable	[73]

**Table 2.** Performance summary of flexible open-system batteries.

Cathode	Capacity	Energy density	Cyclability	Flexibility evaluation	Ref.
LaNiO_3_/NCNT supported on carbon cloth	460 mA h g_Zn_^−1^ at 50 mA g^−1^	581 Wh kg_Zn_^−1^ at 50 mA g^−1^	120 cycles at 50 mA g^−1^ (20 min per cycle)	Bendable	[74]
RuO_2_/NCNT supported on metal/cotton yarn	1981 mA h g_carbon_^−1^ at 320 mA g_carbon_^−1^	None	100 cycles at 200 mA g^−1^ (>600 h)	Bendable	[75]
Fe/Co hydroxide/oxide/NCNT supported on GDL	750 mA h g_Zn_^−1^ at 10 mA cm^−1^	>800 Wh kg_Zn_^−1^ at 10 mA cm^−1^	100 cycles at 5 mA cm^−2^ (5 min per cycle)	None	[76]
NiFe/NCNT supported on nickel foam	None	None	30 cycles at 1 mA cm^−2^ (10 min/cycle)	Bendable	[77]
RuO_2_@CNTs supported on carbon paper	11 400 mA h g^−1^ at 200 mA g^−1^	294.68 Wh kg_device_^−1^ at 100 mA g^−1^	288 cycles at 200 mA g^−1^	Bendable	[78]
Co_3_O_4_-NCNT/SS mesh	∼652.6 mA h g^−1^ at 5 mA cm^−2^, ∼632.3 mA h g^−1^ at 50 mA cm^−2^	847.6 Wh kg^−1^ at 5 mA cm^−2^, 802.6 Wh kg^−1^ at 50 mA cm^−2^	500 h cycle at 25 mA cm^−2^ (20 min/cycle)	Bendable	[79]
NCNTs@SS mesh	9299 mA h g^−1^ at 500 mA g^−1^ (data from coin cell)	None	232 cycles at 500 mA g^−1^ (data from coin cell)	Bendable	[80]
FeNi@NCNT/carbon cloth	None	None	200 cycles at 2 mA cm^−2^ (36 h)	Bendable	[81]
Co-NCNT/carbon nanofiber	11 512.4 mA h g_cathode_^−1^ at 100 mA g_cathode_^−1^ (data from coin cell)	None	130 cycles at 200 mA g^−1^ (data from coin cell)	Bendable	[82]
SWCNT film	∼300 mA h g^−1^ at 1 mA	None	None	Bendable	[86]
CNT@POF film	772.7 mA h g_Zn_^−1^ at 20.0 mA cm^−2^ (data from liquid ZAB)	None	12 cycles at 1 mA cm^−2^ (4 min per cycle)	Bendable	[87]
RuO_2_ and aligned CNT sheets	None	None	30 cycles at 1A g^−1^ (1h per cycle)	Bendable; stretchable	[90]
CNT sheets	7111 mA h g^−1^ at 500 mA g^−1^	2540 Wh kg^−1^	180 cycles at 1000 mA g^−1^	Bendable; stretchable; twistable	[91]
Co_3_O_4_/NCNT composite aerogel films	None	None	20 cycles at 2 mA cm^−2^ (1 h per cycle)	Bendable	[94]
NiCo_2_O_4_@N-OCNT films	None	None	8 cycles charging at 0.5 mA cm^−2^ and discharging at 0.25 mA cm^−2^ (400 s per cycle)	Bendable	[95]
